# Cardiac magnetic resonance imaging of isolated perfused pig hearts in a 3T clinical MR scanner

**DOI:** 10.1186/1532-429X-14-S1-P64

**Published:** 2012-02-01

**Authors:** Andreas Schuster, Amedeo Chiribiri, Masaki Ishida, Geraint Morton, Matthias Paul, Shazia T Hussain, Boris Bigalke, Divaka Perera, Tobias Schaeffter, Eike Nagel

**Affiliations:** 1 Imaging Sciences and Biomedical Engineering, KCL, London, UK

## Summary

We are introducing a novel, refined MR-compatible, explanted, blood-perfused and free-beating pig heart model for translational research within a state of the art 3T XMR environment.

## Background

An isolated perfused pig heart model has recently been proposed for the development of novel methods in standard clinical magnetic resonance (MR) scanners. The original set-up required the electrical system to be within the safe part of the MR-room, which introduced significant background noise. The purpose of the current work was to refine the system to overcome this limitation so that all electrical parts are completely outside the scanner room.

## Methods

Four pig hearts were explanted under terminal anaesthesia from large white cross landrace pigs. All hearts underwent cardiovascular magnetic resonance (CMR) scanning in the MR part of a novel 3T XMR suite. CMR scanning included real-time k-t SENSE functional imaging, k-t SENSE accelerated perfusion imaging and late gadolinium enhancement imaging. Interference with image quality was assessed by spurious echo imaging and compared to noise levels, acquired while operating the electrical parts within the scanner room.

## Results

Imaging was performed successfully in all hearts. The system proved suitable for isolated heart perfusion in a novel 3T XMR suite. No significant additional noise was introduced into the scanner room by our set-up.

## Conclusions

We have substantially improved a previous version of an isolated perfused pig heart model and made it applicable for MR imaging in a state of the art clinical 3T XMR imaging suite. The use of this system should aid novel CMR sequence development and translation into clinical practice.

## Funding

This work was supported by the BHF [RE/08/003 and FS/10/029/28253 to AS, DP, EN], the BRC [BRC-CTF 196 to AS, DP, EN] a European Union Grant [224495 to GM, EN] and the Wellcome Trust and EPSRC Medical Engineering Centre [WT 088641/Z/09/Z to AC, EN].

**Figure 1:**
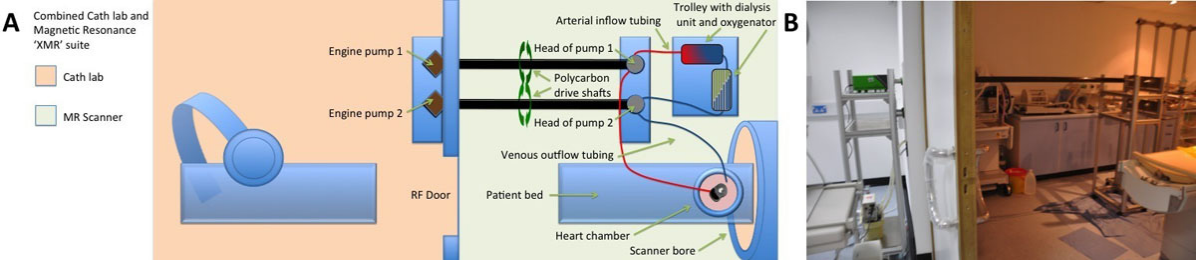
The novel set-up allows the operation of the system through wave guides from the X-Ray room (Cath lab) of the XMR suite. All electrical and magnetic parts remain in the X-Ray room. The pump engines are connected to the custom-made MR compatible pump heads using polycarbon drive shafts through custom made connectors that are fitted in the wave guides.

**Figure 2:**
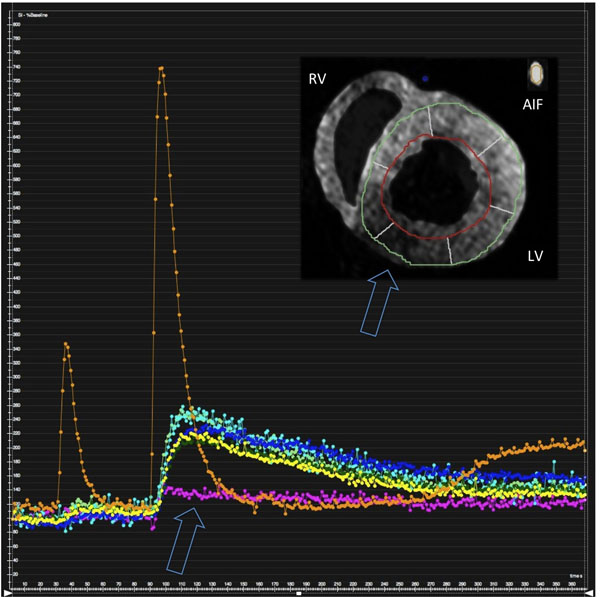
Myocardial first pass dual bolus perfusion imaging showing a perfusion defect in the area of an occluded RCA. Images have been segmented for better visibility. LV left ventricle, RV right ventricle, AIF arterial input function (taken from the inflow tubing).

